# Effect of extracorporeal shock wave therapy for rotator cuff injury: Protocol for a systematic review and meta-analysis

**DOI:** 10.1371/journal.pone.0301820

**Published:** 2024-05-08

**Authors:** Xiali Xue, Qingfa Song, Xinwei Yang, Amila Kuati, Hao Fu, Guoqing Cui

**Affiliations:** 1 School of Sports Medicine and Health, Chengdu Sport University, Chengdu, Sichuan Province, China; 2 Department of Sports Medicine, Institute of Sports Medicine of Peking University, Peking University Third Hospital, Beijing, China; 3 Chengdu University of Traditional Chinese Medicine, Chengdu, Sichuan Province, China; 4 Department of Rehabilitation, Peking University Third Hospital, Beijing, China; Universiti Malaya, MALAYSIA

## Abstract

**Background:**

Rotator cuff injury (RCI) is a common musculoskeletal ailment and a major cause of shoulder pain and limited functionality. The ensuing pain and restricted movement significantly impact overall quality of life. This study aims to systematically review the effects of extracorporeal shock wave therapy (ESWT) on RCI.

**Methods:**

This protocol follows the Preferred Reporting Items for Systematic Reviews and Meta-Analyses Protocols. A literature search, spanning inception to November 1, 2023, will include databases such as PubMed, Web of Science, the Cochrane Library, Scopus, MEDLINE, EMBASE, EBSCO, and China National Knowledge Infrastructure (CNKI) to identify ESWT studies for RCI treatment. Excluding retrospectives, bias risk will be assessed with the Cochrane tool. Two researchers will independently screen, extract data, and evaluate bias risk. Revman 5.3 software will be used for data analysis.

**Results:**

This study aims to objectively and comprehensively evaluate the effectiveness and safety of randomized controlled trials of ESWT in the treatment of RCI, and analyze in detail the effect of ESWT in the treatment of RCI. Results will be analyzed using the Pain Visual Analogue Scale (VAS), Constant-Murley score, University of California Los Angeles score (UCLA), and American Shoulder and Elbow Surgeons form (ASES). If applicable, subgroup analysis will also be performed to divide patients into groups according to the energy level of ESWT, the time of intervention, and the degree of tearing of RCI. Finally, the results are submitted for publication in a peer-reviewed journal.

**Discussion and conclusion:**

There is existing evidence suggesting that ESWT may contribute to the amelioration of pain and functional limitations associated with Rotator Cuff Injury (RCI). This systematic review aims to update, consolidate, and critically evaluate relevant evidence on the effects of ESWT for RCI. The anticipated outcomes may serve as a valuable reference for clinical ESWT practices, covering treatment methods, timing, and intensity. Moreover, this review aspires to provide high-quality evidence addressing the impact of ESWT on RCI-related pain. Simultaneously, the findings of this systematic review are poised to offer guidance to clinicians and rehabilitation therapists. This guidance is intended to enhance the management of pain and functional impairments experienced by individuals with RCI, ultimately leading to improvements in their physical well-being.

**Trial registration:**

**Protocol registration number** CRD42023441407. https://www.crd.york.ac.uk/prospero/display_record.php?ID=CRD42023441407.

## Introduction

Rotator cuff injury (RCI) represents a prominent source of shoulder pain and functional impairment, affecting as many as 1 in 3 individuals during their lifetime [1]. The prevalence of RCI is notably higher, with 25% of individuals aged over 60 years and a striking 50% among those aged over 80 years [2]. These injuries often result in limitations in daily activities and shoulder joint mobility [3]. The etiology of RCI is intricate, involving a multifaceted interplay of factors, and its pathogenesis is not fully understood. Common contributors include aging, overuse, mechanical trauma, smoking, and familial predisposition, with studies suggesting a genetic component in rotator cuff disease development [4]. RCI typically results from two primary mechanisms: injury and degeneration. Acute tears often stem from traumatic injury, while chronic shoulder pain primarily emerges from recurrent impingement of the rotator cuff against the acromion. Initial symptoms include local cuff edema and hemorrhage, progressing to tendinitis with localized fibrosis. [5,6]. Prolonged exposure to predisposing factors can eventually culminate in a rotator cuff tear [7].

The management of RCI is primarily categorized into surgical and non-surgical approaches [8]. Existing evidence indicates that both physical therapy and surgical interventions can yield significant improvements in patient-reported outcomes, particularly in symptomatic individuals with small-to-moderate full-thickness RCI [6,9]. Extracorporeal Shock Wave Therapy (ESWT) has gained widespread acceptance as a non-invasive treatment modality for a range of bone and muscle injuries [10]. ESWT is associated with a spectrum of biological effects, encompassing tissue regeneration, wound healing, angiogenesis, bone remodeling, and anti-inflammatory responses [11]. Its mechanical actions include influencing various cellular structures such as mitochondria, endoplasmic reticulum, and intracellular vesicles, as well as enzymatic processes. The cumulative effect of these responses results in an enhancement of the overall healing process [12]. ESWT plays a crucial role in loosening adhesive tissues, expediting skeletal muscle injury recovery, and mitigating internal inflammation, resulting in a pronounced analgesic effect. In recent years, shock wave therapy has shown remarkable efficacy in treating RCI, becoming a preferred non-invasive option in rehabilitation, especially for alleviating chronic pain and tendinosis, given its high safety profile. ESWT has established itself as a valuable therapeutic approach for managing RCI, offering a compelling treatment alternative [13].

### Objectives

Currently, the effectiveness of ESWT in the treatment of RCI remains a subject of debate within the medical community. Some studies have reported significant benefits of extracorporeal shock waves in terms of pain reduction, functional improvement, and tissue repair promotion among RCI patients [14,15]. Conversely, other studies have arrived at contradictory conclusions [16,17]. Systematic reviews addressing the impact of extracorporeal shock waves on shoulder pain and function in RCI patients are relatively scarce, and the most recent research findings are yet to be incorporated.

This study is designed to systematically review and conduct a meta-analysis to assess the influence of ESWT on shoulder pain alleviation and functional recovery in individuals afflicted with RCI. Through a comprehensive assessment of clinical efficacy and underlying scientific rationale, our goal is to provide valuable insights and serve as a reference for future research endeavors in this field.

## Methods

### Study protocol

This systematic review protocol follows the guideline recommended in Preferred Reporting Items for Systematic Review and Meta-Analysis (PRISMA-P) [18] (see S1 Checklist) and has been registered in the PROSPERO database (International Prospective Register of Systematic Reviews) with registration number CRD42023441407.

### Eligibility criteria

#### Inclusion criteria

*Study population*. Adult patients (18 years of age and older, includes athletes in a variety of upper extremity sports.) with RCI will be included, consistent with clinical or radiographic findings, regardless of race, nationality, or course of disease. The umbrella term RCI includes a variety of shoulder joint diseases that affect the rotator cuff structure, such as rotator cuff tendinitis, and partial tear of rotator cuff.

*Intervention*. The experimental group was treated with ESWT.

*Comparator*. The control group was treated with a placebo ESWT or other treatments (Such as pharmacotherapy, or other comparable interventions).

*Outcome*. The main outcome indicator is the Visual Analogue Scale/Score (VAS), Secondary outcome indicators are the Constant-Murley score (CMS), University of California Los Angeles score (UCLA), Range of Motion (ROM), American Shoulder and Elbow Surgeons form (ASES) and Total effective rate (TER).

*Study design*. All clinical randomized controlled trials (RCTs) of ESWT for the treatment of RCI will be included in this review.

#### Exclusion criteria

The following types of studies will be excluded from the analysis:

1. Animal experiments.
2. Studies where comparable between the intervention and control groups is lacking.
3. Letters, reviews, case reports, conference abstracts, and comments.

### Search strategy

The literature search will be conducted on the following databases from inception to November 1, 2023: PubMed, Web of Science, the Cochrane Library, Scopus, MEDLINE, EMBASE, EBSCO, and China National Knowledge Infrastructure (CNKI) will be systematically reviewed to identify potential studies investigating the efficacy of ESWT in treating RCI. The search strategy will employ a combination of subject headings and free-text terms, Boolean operators (AND or OR), and tailored search strategies adapted to the specific requirements of each database. Search terms included “Rotator cuff injuries”, “Cuff Injury, Rotator”, “Rotator Cuff Tear”, “Rotator Cuff Tendinitis”, “Extracorporeal shock wave therapy”, “ESWT”, “Physical therapy modalities”, “Physical therapy”, “Randomized controlled trial,” “Controlled clinical trial,” “Randomized.” Using PubMed as an example, the specific retrieval strategy is outlined in Table 1. Additionally, to ensure comprehensive coverage and minimize the risk of missing any eligible randomized controlled trials, references from clinical registration websites, relevant conference presentations, and pertinent publications will be meticulously searched.

**Table 1:** Search strategy for the PubMed database.

# No	Searches
**#1**	“Rotator Cuff Injuries” [Mesh]
**#2**	“Cuff Injury, Rotator”[Title/Abstract] OR “Injury, Rotator Cuff”[Title/Abstract] OR “Rotator Cuff Injury”[Title/Abstract] OR “Rotator Cuff Tears”[Title/Abstract] OR “Rotator Cuff Tear”[Title/Abstract] OR “Tear, Rotator Cuff”[Title/Abstract] OR “Tears, Rotator Cuff”[Title/Abstract] OR “Rotator Cuff Tendinosis”[Title/Abstract] OR “Rotator Cuff Tendinoses”[Title/Abstract] OR “Tendinoses, Rotator Cuff”[Title/Abstract] OR “Tendinosis, Rotator Cuff”[Title/Abstract] OR “Rotator Cuff Tendinitis”[Title/Abstract] OR “Rotator Cuff Tendinitides”[Title/Abstract] OR “Tendinitis, Rotator Cuff”[Title/Abstract] OR “Glenoid Labral Tears”[Title/Abstract] OR “Glenoid Labral Tear”[Title/Abstract] OR “Labral Tear, Glenoid”[Title/Abstract] OR “Labral Tears, Glenoid”[Title/Abstract] OR “Tear, Glenoid Labral”[Title/Abstract]
**#3**	**#**1 OR **#**2
**#4**	“Extracorporeal Shockwave Therapy” [Mesh]
**#5**	“Extracorporeal Shockwave Therapies”[Title/Abstract] OR “Shockwave Therapies, Extracorporeal”[Title/Abstract] OR “Shockwave Therapy, Extracorporeal”[Title/Abstract] OR “Therapy, Extracorporeal Shockwave”[Title/Abstract] OR “Shock Wave Therapy”[Title/Abstract] OR “Shock Wave Therapies”[Title/Abstract] OR “Therapy, Shock Wave”[Title/Abstract] OR “Extracorporeal Shock Wave Therapy”[Title/Abstract] OR “Extracorporeal High-Intensity Focused Ultrasound Therapy”[Title/Abstract] OR “Extracorporeal High Intensity Focused Ultrasound Therapy”[Title/Abstract] OR “HIFU Therapy”[Title/Abstract] OR “HIFU Therapies”[Title/Abstract] OR “Therapy, HIFU”[Title/Abstract] OR “High-Intensity Focused Ultrasound Therapy”[Title/Abstract] OR “High Intensity Focused Ultrasound Therapy”[Title/Abstract]
**#6**	**#**4 OR **#**5
**#7**	“Randomized Controlled Trial” [Mesh]
**#8**	“Randomized Controlled Trial” [Title/Abstract] OR “Controlled Clinical Trials, Randomized” [Title/Abstract] OR “Clinical Trials, Randomized”[Title/Abstract] OR “Trials, Randomized Clinical”[Title/Abstract] OR “Clinical trial”[Title/Abstract] OR “Clinical trials”[Title/Abstract]
**#9**	**#**7 OR **#**8
**#10**	**#**3 AND **#**6 AND **#**9

### Selection process

**Fig 1:**
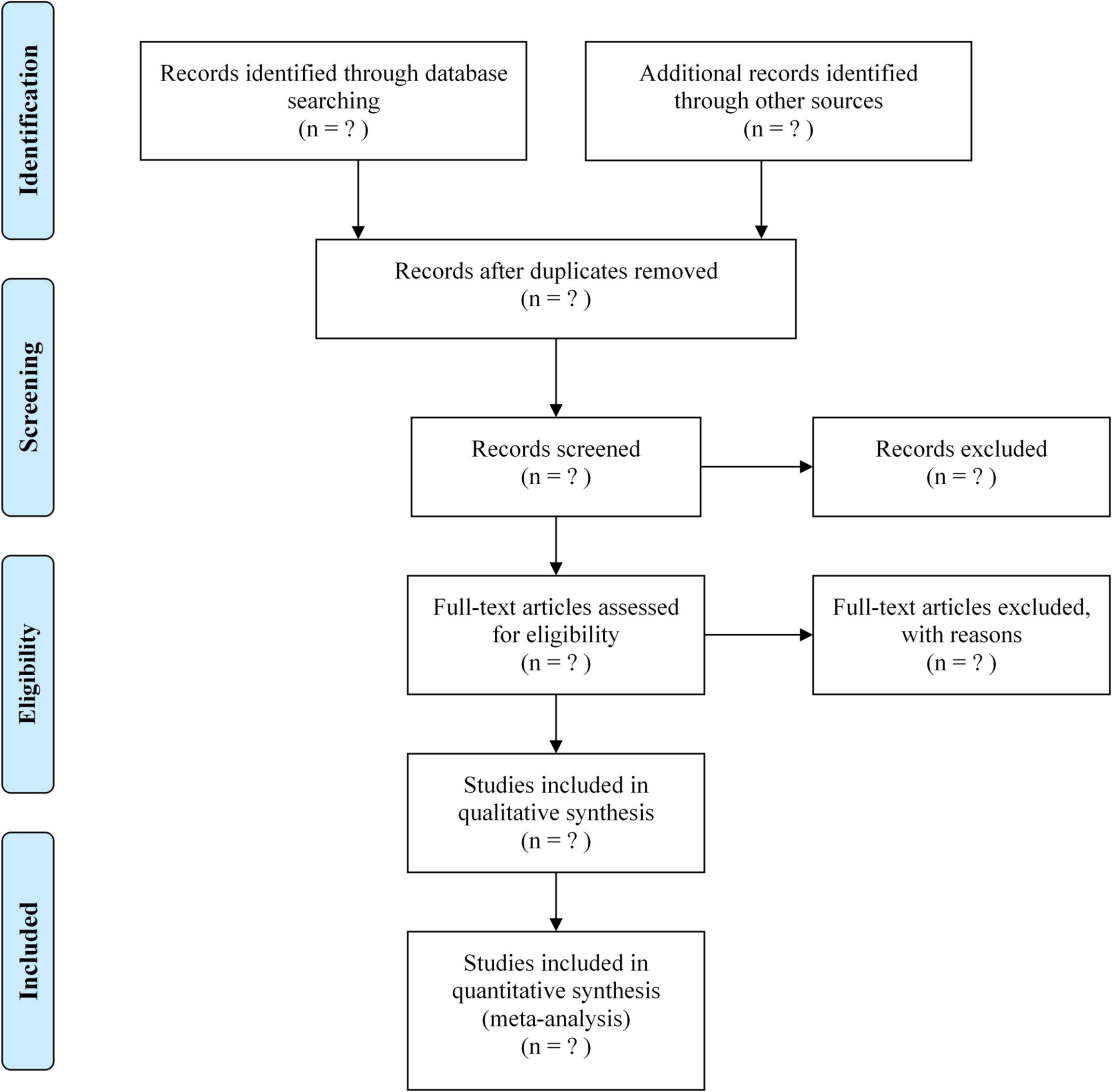
Flow diagram of the study selection process.

First, the retrieved documents will be imported into Endnote X9, and duplicates will be removed. Second, before the screening of studies, the research team will engage in discussions to establish and define the screening criteria collaboratively. Subsequently, two experienced reviewers (XXL and SQF), will independently undertake the screening and assessment of the titles and abstracts of each study following the predefined criteria. Studies that do not meet the qualification criteria will be excluded, and the remaining full-text articles will be reviewed and screened based on the previously established criteria. In cases where discrepancies or disagreements arise, resolutions will be reached through discussions involving the corresponding author (CGQ). All stages of the selection process will adhere to the guidelines presented in the PRISMA flow chart. The literature screening process is illustrated in Fig 1.

### Data collection process

Two researchers (XWY and HF) will independently extract data in the future. Data will be independently collected according to a pre-designed data collection form, and the following information will be extracted using a spreadsheet: first author information, publication date, year of publication, country, study design, sample size, basic characteristics of patients (overuse or mechanical trauma), intervention in treatment and control groups measures, primary and secondary results. If the data shown above is incomplete in the future, we will contact the article author. Any future disagreements regarding data extraction will be resolved through discussion with a third researcher (GQC).

### Study risk of bias assessment

Two researchers (XXL and SQF) independently assessed the methodological quality of each reviewed study by using the Cochrane Risk of Bias tools 2.0 (ROB 2.0) [19] to assess the risk of bias in randomized trials, and differences were resolved through discussion, or a third researcher (CGQ) was consulted if consensus could not be reached. The methodological quality of studies will be assessed by randomization process, Deviation from intended interventions, Missing outcome data, Measurement of the outcome, and Selection of the reported result. Each of these domains will be assessed as Low risk, High risk, and Some concerns according to the ROB 2.0.

### Management of missing data

For studies with missing data, we will attempt to contact the corresponding author of the article to obtain the missing information. If contact cannot be obtained, we will delete or use the available data to complete our analysis or use descriptive analysis, as appropriate.

### Assessment of reporting biases

When more than 10 studies were included, funnel plots were used to determine whether reporting bias existed. If the image is not clear, evaluate and analyze it by using Egger’s test.

### Subgroup analysis

If significant heterogeneity exists between study results, we will perform a subgroup analysis. Subgroup analysis will be performed based on different doses of ESWT, duration of intervention, and severity of rotator cuff injury.

### Sensitivity analysis

When sufficient studies are available, sensitivity analyses are required for possible low-quality studies. Tests with quality defects were excluded to check the stability of the final results.

### Grading the quality of evidence

This study will be evaluated using the GRADE system in the future, with evidence categorized into four levels: very low, low, medium, or high. Ultimately, the results will be presented in tabular form in the final publication [20].

### Statistical analysis

The meta-analysis will be conducted using RevMan version 5.3. Standardized mean difference (SMD) and 95% confidence interval (CI) will be employed for continuous variables, while the odds ratio (OR) will be used for categorical variables in the pooled analysis. The assessment of statistical heterogeneity between studies will be done using P and I^2^ values. If there is no significant heterogeneity between study results, a fixed-effects model will be applied for meta-analysis. In case a certain degree of heterogeneity exists, a random effects model will be considered. When significant clinical heterogeneity exists, subgroup or sensitivity analysis or only descriptive analyses will be utilized.

### Ethics and dissemination

The present study will use published data and does not require ethics approval.

## Discussion

RCI stands out as one of the most common musculoskeletal degenerative conditions linked to the aging process. The etiology of RCI is notably multifaceted, involving various factors that result in a diverse range of clinical treatment options. Determining the optimal approach for treatment remains an area of ongoing investigation. Surgical interventions, while effective, come with inherent risks such as secondary infections. On the other hand, conservative drug-based therapies are susceptible to a range of adverse reactions, a concern particularly relevant among elderly individuals who show an elevated susceptibility to complications related to medication [21]. Given these considerations, the selection of a safer and more efficacious treatment strategy for elderly patients grappling with RCI becomes imperative. ESWT, an increasingly adopted non-invasive intervention, has emerged as a viable option in the recent management of RCI. The principal mechanistic actions underlying ESWT encompass the following: ESWT capitalizes on the mechanical effect generated through local mechanical vibrations and cavitation, inducing alterations in human tissues and cellular structures. It facilitates the expansion of blood vessels, thereby promoting the regeneration of tendons and soft tissues [22]; Furthermore, ESWT exerts an inhibitory influence on pain receptors, employing high-frequency pulses to disrupt the transmission of pain signals. This intervention enhances water and electrolyte circulation and augments the metabolic processes within the treatment region, leading to the amelioration of local inflammation. Consequently, it reduces load-bearing constraints and alleviates pain, thus enhancing shoulder joint function and increasing the ROM of the shoulder joint [23]. ESWT is widely employed to alleviate pain caused by various types of shoulder cuff injuries, including acute injuries and chronic conditions. Its non-invasive nature makes it a relatively safe treatment option suitable for patients of different ages and health conditions. As a conservative treatment choice, it avoids the risks and complexities associated with surgery. This positions it as an alternative in the management of shoulder cuff injuries, particularly for patients who prefer to avoid surgery or are not suitable candidates for surgical interventions.

The primary objectives of RCI treatment encompass effective pain mitigation and the preservation of functional capabilities. Once pain is successfully managed, maintaining and enhancing functionality typically entails a regimen of exercises designed to expand the range of motion (ROM) and fortify the rotator cuff muscles. Notably, ESWT demonstrates the capacity to effectively ameliorate adhesion tissue and alleviate spasms within soft tissues, thereby facilitating an increase in the ROM of the shoulder joint. Various etiologies of RCI including rotator cuff tendonitis, partial rotator cuff tears, adhesive capsulitis, subscapular bursitis, and complex regional pain syndrome are thought to lead to antifibrotic, anti-inflammatory, and pain-modulating effects [24]. Since RCI includes an inflammatory response, ESWT can eliminate inflammatory factors in the patient’s body, relieve pain, promote the early recovery of shoulder joint function, and improve the curative effect. Ko et al. employed a single session of high-energy ESWT with long-term follow-up and demonstrated its efficacy in improving the functional outcome of rotator cuff lesions accompanied by shoulder stiffness. These findings suggest that ESWT represents a simple, effective, and non-invasive treatment option for such conditions [25]. Similar results were also observed in other studies, with significant improvement in pain reduction and shoulder function in the ESWT group compared with the sham group [26,27]. In addition, the adverse effects of ESWT were dose-dependent and usually limited to temporary increases in pain and local reactions, such as swelling, erythema, petechiae, or small hematomas, and no serious adverse events were reported [28].

In a clinical trial conducted by Duymaz et al., the experimental group underwent a combined treatment approach consisting of ESWT in conjunction with conventional Physical Therapy (PT), whereas the control group received conventional PT alone. The assessment of patients’ pain levels was conducted utilizing the VAS both before and after the intervention. Remarkably, the experimental group exhibited notable improvements in VAS scores. Specifically, patients receiving ESWT treatment experienced a remarkable enhancement in shoulder function, with an impressive 43% improvement compared to the control group [29]. Li et al. conducted a comprehensive study in which they compared ESWT with a placebo intervention. The study employed various measurement tools, including the Numeric Pain Rating Scale, CMS, and other relevant metrics. These assessments were carried out at the 4th and 8th weeks of the study. The results yielded from the ESWT group demonstrated significant improvements, underscoring the potential efficacy of ESWT in managing shoulder-related conditions [30].

However, a study by Kvalvaag et al. investigated the effects of placebo ESWT compared to ESWT in combination with supervised exercise for patients suffering from subacromial pain syndrome. The study aimed to identify predictors of pain, disability, and work status within this patient cohort. After one year of thorough follow-up, the research did not reveal any statistically significant differences between the two treatment groups concerning primary or secondary outcome measures. These findings suggest that ESWT did not provide additional benefits when combined with supervised exercise within this patient population, whether in the short, medium, or long term. Further analysis indicated that negative outcome expectations, frequent use of pain medication, not being employed at the study baseline, single marital status, lower self-reported general health, and limited participation in supervised exercise classes were predictive of poorer SPADI outcomes after one year [31]. In a study comparing placebo ESWT to ESWT combined with RCI treatment, Kolk et al. observed noteworthy improvements in VAS, CMS, and Simple Shoulder Test scores in both groups at the 3- and 6-month post-treatment assessments. However, it is important to note that the low-dose ESWT applied as a placebo did not appear to effectively alleviate symptoms in patients suffering from chronic rotator cuff tendinitis. Consequently, the study did not establish a clear therapeutic advantage of ESWT in patients with shoulder tendonitis [32]. These findings support the results of a previous study conducted by Schmitt et al., which similarly reported that ESWT did not lead to significant improvements in CMS, SPADI scores, or pain levels among patients with noncalcified cuff tendonitis [33]. Additionally, the question of whether the self-limiting nature of RCI justifies the utilization of ESWT for long-term outcomes remains a topic open for further discussion and examination [34].

## Prospects

RCI treatment is an individualized process, requiring tailored plans based on patients’ specific conditions. Future research will emphasize individualized treatment methods, where medical experts will comprehensively consider factors such as the patient’s condition, symptom severity, and physical well-being. They will then choose the most appropriate treatment method based on the latest clinical guidelines and research results. Additionally, ESWT may be combined with other modalities, such as physical therapy, medication, or surgery, for enhanced outcomes. Integrating different treatments can maximize function and pain relief for patients. With ongoing advancements in medical technology, ESWT equipment may be refined for increased precision, efficiency, and safety, offering more treatment options for different types of RCI. Beyond treatment, future research will focus on the prevention and rehabilitation of RCI. Strategies like strengthening exercises, posture improvement, and avoiding overuse may help reduce the incidence of RCI. Simultaneously, optimizing rehabilitation programs will enhance their effectiveness and prevent recurrence.

## Limitations

However, this systematic review has certain limitations. Language constraints dictated our search to encompass only Chinese and English literature. Within the included studies, variations in the brand, technique, placement, and size of the ESWT probe may exist, potentially contributing to clinical heterogeneity. Disparities in the timing and methodology of ESWT interventions among patients also pose a potential source of clinical heterogeneity.

## Conclusions

This systematic evaluation represents the inaugural attempt to comprehensively assess the effectiveness and safety of ESWT in the treatment of RCI. The findings of this study will be instrumental in deriving meaningful conclusions regarding the impact of ESWT on RCI. The ultimate goal is to make valuable contributions to the field of RCI prevention and treatment.

Any amendments made to the Protocol will be reflected in PROSPERO. Papers will be submitted to peer-reviewed orthopedic or rehabilitation journals. Opportunities to present at research conferences will also be sought. If possible, we will also disseminate it at relevant academic conferences.

## Supporting information

S1 Checklist PRISMA-P (Preferred Reporting Items for Systematic Review and Meta-Analysis Protocols) 2015 checklist: Recommended items to address in a systematic review protocol. (DOC)
